# HMGB-1 as a Novel Predictor of Disease Severity and Prognosis in Patients with Hemorrhagic Fever with Renal Syndrome

**DOI:** 10.1155/2015/696248

**Published:** 2015-04-06

**Authors:** Hong Du, Jing Li, Haitao Yu, Jianqi Lian, Ye Zhang, Ying Zhang, Xuefan Bai, Pingzhong Wang

**Affiliations:** Center of Infectious Diseases, Tangdu Hospital, Fourth Military Medical University, Xi'an 710038, China

## Abstract

*Objective*. To examine the predictive capacity of the high mobility group box protein-1 (HMGB-1) for disease severity and prognosis of hemorrhagic fever with renal syndrome (HFRS). *Methods*. One hundred and five HFRS patients and 28 controls were studied. The concentrations of HMGB-1 in the blood were measured with a commercially available ELISA. The levels of white blood cells (WBC), platelets (PLT), hematocrit (HCT), albumin (ALB), blood urea nitrogen (BUN), serum creatinine (Scr), and uric acid (UA) were routinely tested in the same time frame. *Results*. The levels of HMGB-1 increased with the severity of the disease (*P* < 0.001). HMGB-1 was positively correlated with WBC and BUN and negatively correlated with PLT, ALB, and UA (*P* < 0.001). HMGB-1 showed statistical significance for predicting prognosis (AUC = 0.800, *P* < 0.001). The sensitivity and specificity of HMGB-1, WBC, PLT, and ALB used in combination for predicting outcome were better than those of single analyses (AUC = 0.892, *P* < 0.001). *Conclusions*. HMGB-1 can be considered a novel biomarker for severity and outcome in patients with HFRS. The use of HMGB-1, WBC, PLT, and ALB in combination to predict the outcome in patients with HFRS exhibited an acceptable level of diagnostic capability.

## 1. Background

Hemorrhagic fever with renal syndrome (HFRS) is a rodent-borne disease that is caused by Hantavirus (HV), with major clinical characteristics of fever, hemorrhage, hypotension, and renal damage [1, 2]. The typical disease progresses through five phases: febrile, hypotensive, oliguric, diuretic, and convalescent [3, 4]. China is the most severe endemic area of HFRS in the world, with a high incidence rate in the last ten years [5]. Xi'an city is the center of Shaanxi province and one of the most severely affected regions in China. The pathogen that causes HFRS having been discovered in the city is Hantaan virus, the major serotype of HV carried by the rodents* Apodemus agrarius* [6]. Furthermore, the HFRS patients in this district display more severe manifestations, usually accompanied with refractory shock, acute respiratory distress syndrome (ARDS), encephalopathy, disseminated intravascular coagulation (DIC), and multiple organ dysfunction syndrome (MODS), which leads to a greatly increased fatality rate.

There is no specific antiviral therapy for HFRS, and the identification of a single laboratory parameter that is routinely tested clinically to evaluate severity and predict the outcome remains challenging because of the nonspecific, complicated clinical courses and the pathophysiology of the disease. Nevertheless, an accurate early determination of disease severity and the investigation of new biomarker are still very important to timely and systematic treatment. In this study, we detected the concentrations of the high mobility group box protein-1 (HMGB-1) in HFRS patients and explored its predictive value on the disease severity and prognosis.

## 2. Materials and Methods

### 2.1. Ethics Statement

The perspective study was approved by the Institutional Review Board of Tangdu Hospital. Before inclusion, the patients were informed about the objectives of this study; they or their direct relatives agreed and signed the informed consent form so that blood samples and medical records could be obtained.

### 2.2. Study Participants

One hundred and five patients with HFRS that were treated at our center between October 2011 and December 2012 were randomly enrolled in this study. The demographic characteristics of the patients were collected from medical records. Patients who had other kidney diseases, diabetes, cardiovascular disease, hematological disease, autoimmune disease, viral hepatitis, and other liver diseases were excluded.

The diagnosis of HFRS was made based on the positive enzyme linked immunosorbent assay (ELISA) result for specific IgM and IgG antibodies against Hantaan virus in acute phase serum. The assay was performed using IgG/IgM capture ELISA kits and was analyzed via a multifunctional autoanalyzer (BIORAD-680, United States).

According to the HFRS criteria of clinical classification [7], the severity of HFRS was classified into four types: (1) mild, defined as patients who had kidney injury without oliguria and hypotension; (2) moderate, defined as patients who had uremia, effusion (bulbar conjunctiva), hypotension, hemorrhage (skin and mucous membranes), and AKI with typical oliguria; (3) severe, defined as patients who had severe uremia, effusion (bulbar conjunctiva and either peritoneum or pleura), hemorrhage (skin and mucous membranes), hypotension, and AKI with oliguria (urine output of 50–500 mL/day) for ≤5 days or anuria (urine output of <100 mL/day) for ≤2 days; and (4) critical, defined as patients who usually had one or more of the following complications compared with the severe patients: refractory shock (≥2 days), visceral hemorrhage, heart failure, pulmonary edema, brain edema, severe secondary infection, and severe AKI with oliguria (urine output of 50–500 mL/day) for >5 days or anuria (urine output of <100 mL/day) for >2 days. Considering the clinical conditions that a majority of the survival patients had been discharged before the convalescent phase and the degree of acute kidney injury (AKI) that was still severe during the early stage of the diuretic phase, the acute stage was defined as the period that included the febrile, hypotensive, and oliguric phases and the early three days of the diuretic phase in this study, and the convalescent stage was defined as the diuretic and convalescent phase except the early three days of the diuretic phase. Furthermore, the patients were followed up until 28 days after discharge, and the prognosis (death) in this study was defined as patient death during hospitalization or within the 28 days following discharge.

### 2.3. Blood Samples and Detection

Ninety-three venous blood samples were drawn randomly from the patients during the acute stage, and 78 samples were drawn randomly during the convalescent stage. Twenty-eight blood samples from healthy subjects were obtained as controls. All of the samples were stored in EDTA tubes and were centrifuged at 2,000 rpm for 10 min at 4°C within 2 hours after drawing. The plasma supernatant was pipetted carefully and transferred to polypropylene tubes and then stored at −80°C prior to HMGB-1 analysis.

HMGB-1 levels were measured with commercially available ELISA kits (Quantikine, XiTang, Inc., Shanghai, China) and were tested using a multifunctional autoanalyzer (BIORAD-680, United States) according to the manufacturer's instructions. Each sample was detected twice and the sensitivity of the minimum concentration of HMGB-1 was below 0.3 ng/mL.

Seven laboratory parameters including white blood cells (WBC), platelets (PLT), hematocrit (HCT), albumin (ALB), blood urea nitrogen (BUN), serum creatinine (Scr), and uric acid (UA) were routinely tested using autoanalyzers (Sysmex XT-4000i, Japan; Hitachi 7600-100, Japan). All the laboratory parameters mentioned above and HMGB-1 were measured in the same time frame.

### 2.4. Statistical Analysis

Statistical analysis was performed using SPSS 17.0 software (SPSS Inc., Chicago, IL, USA). Tables were created using Excel 2003 (Microsoft), and figures were created using GraphPad Prism 5 (GraphPad Software, San Diego, CA). Continuous variables are presented as the mean ± SD and were analyzed by Kolmogorov-Smirnov's test for normal distribution and by Levene's test for the homogeneity of variance. The variables among the four types were compared by SNK test for normally distributed variables. The nonnormally distributed variables are presented as medians with interquartile ranges and were compared by the nonparametric Kruskal-Wallis *H* test. The Nemenyi Rank test was used to compare the differences among the four types. The frequencies and percentages are given for qualitative variables, and the differences among the four types were tested using Pearson's chi-square test. Pearson's correlation coefficient was used to determine the relationship between HMGB-1 and the laboratory parameters as mentioned above. The predictor values of HMGB-1 for disease prognosis were tested using receiver operating characteristic (ROC) curves and quantified by calculating the area under the ROC curve (AUC) and the 95% confidence interval (CI). A two-tailed *P* < 0.05 was considered statistically significant.

## 3. Results

### 3.1. Clinical Typing and Demographic Characteristics for Patients with HFRS

Of the enrolled patients, 19 cases were classified as mild, 25 cases were classified as moderate, 27 cases were classified as severe, and 34 cases were classified as critical according to the HFRS criteria of clinical classification. Twelve critical individuals died during the acute stage with a hospital mortality rate of 11.42%. There was no significant difference in the sex or age distribution among the groups (*P* > 0.05) (Table 1).

### 3.2. Levels of HMGB-1 in Patients with HFRS

**Figure 1:**
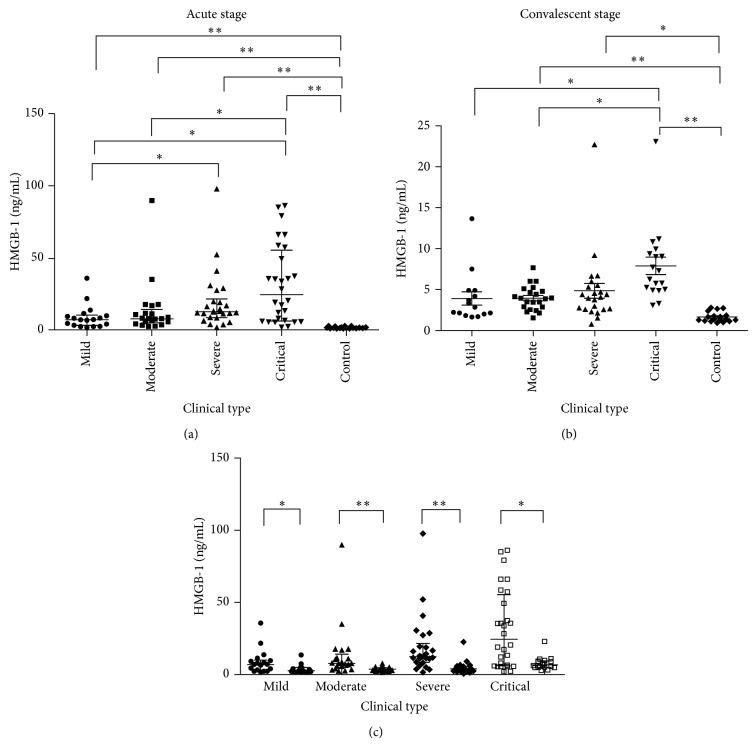
Levels of HMGB-1 during the clinical course in patients with HFRS. The concentrations of HMGB-1 were presented as medians with IQR and were compared by the Nemenyi Rank test among the five groups ((a) and (b)). The concentrations of HMGB-1 were presented as medians with IQR and were compared by a Mann-Whitney *U* test for the acute stage and convalescent stage (c). ^∗^ *P* < 0.05; ^∗∗^ *P* < 0.001.

The duration from disease onset to sample collection among the groups was not significantly different (*P* > 0.05) (Table 2). The levels of HMGB-1 in the patients from the acute stage were significantly higher than control (*P* < 0.001) and were increased with the severity of the disease. The levels of HMGB-1 in the patients in the convalescent stage were higher than the control group, except for the mild-type group (*P* < 0.05); the HMGB-1 levels of the critical-type group were higher compared with the mild- and moderate-type groups (*P* < 0.05) (Table 3, Figure 1).

### 3.3. Pearson Correlation Analysis and ROC Curves

**Figure 2:**
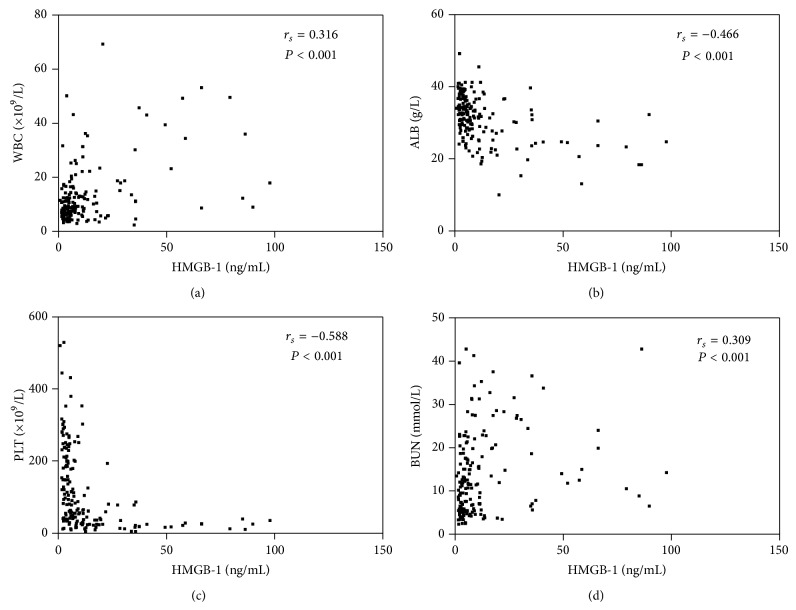
Correlation between HMGB-1 and WBC (a), ALB (b), PLT (c), and BUN (d) in patients with HFRS. HMGB-1, high mobility group box protein-1; WBC, white blood cells; PLT, platelets; ALB, albumin; BUN, blood urea nitrogen.

Pearson correlation analysis revealed that HMGB-1 was positively correlated with WBC and BUN and was negatively correlated with PLT, ALB, and UA (*P* < 0.001) (Table 4, Figure 2).

**Figure 3:**
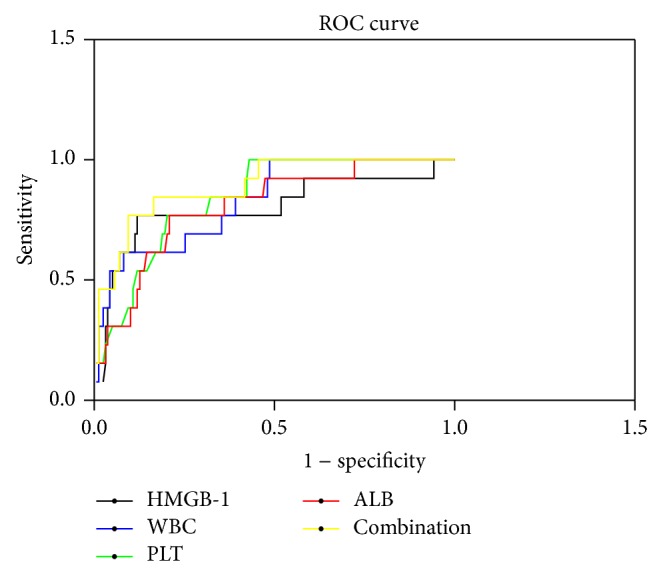
Use of HMGB-1, WBC, PLT, and ALB in combination to predict prognosis in patients with HFRS by ROC analysis. ROC, receiver operating characteristic; HMGB-1, high mobility group box protein-1; WBC, white blood cells; PLT, platelets; ALB, albumin.

ROC analysis revealed that HMGB-1 showed statistical significance for predicting prognosis with the area under the curve (AUC) equal to 0.800 (95% CI: 0.645–0.955, *P* < 0.001). The sensitivity and specificity of the HMGB-1, WBC, PLT, and ALB in combination for predicting outcome were better than with individual analysis (AUC = 0.892, *P* < 0.001) (Table 5, Figure 3).

## 4. Discussion

This is the first study to explore the predictive capacity of HMGB-1 for the severity and prognosis of HFRS. HMGB1 was discovered as a nuclear DNA-binding protein 30 years ago. It can be released by activated monocytes, macrophages, neutrophils, and platelets and, in turn, mediates inflammation and enhanced cell motility [8]. It has been proven that the lipopolysaccharide (LPS) that is released by gram-negative bacteria and proinflammatory factors such as TNF-*α*, IL-1, and IFN-*γ* can positively stimulate the secretion of HMGB-1; extracellular HMGB-1 can be secreted passively by necrotic rhagiocrine cell [9]. Extracellular HMGB-1 can be considered an effective mediator that further induces and promotes the inflammatory response [10, 11]. As a key late-phase proinflammatory cytokine [12], HMGB-1 participates in the physiopathological course of sepsis [13] and has become an important target for the prevention and treatment of sepsis.

In the past ten years, only a minority of research focused on observing the predictive role of HMGB-1 on disease severity and prognosis in patients with sepsis, including different findings. Sundén-Cullberg et al. [14] found increased HMGB-1 serum levels in patients with sepsis or septic shock in a prospective study with 33 septic patients. They did not find a significant difference between the septic and septic shock patients, and HMGB-1 was not found to be correlated with the prognosis of sepsis. In another study that was recently published, Gibot et al. [15] reported that the septic shock nonsurvivors had higher HMGB-1 concentrations than survivors on day 3 but not at the time of hospital admission. They also found that the HMGB-1 concentrations on day 3 could be beneficial to predicting the prognosis of the septic shock patients, and HMGB-1 was closely correlated with the degree of multiple organ dysfunctions. In one study, Angus et al. [16] found that the circulating blood of patients with pneumonia and with pneumonia combined with serious sepsis demonstrated greatly elevated levels of HMGB-1, while there was no significant difference between the two groups. They also found that the HMGB-1 concentrations in nonsurvivors were lower compared with the survivors.

Considering that the role of a single laboratory parameter that is routinely tested clinically for severity evaluation and outcome prediction is still challenging, we observed levels of high mobility group box protein-1 (HMGB-1) in different clinical phases of HFRS and explored its predictive value on disease severity and prognosis. Unlike the results from the research in sepsis patients as mentioned above, this study demonstrated that HMGB-1 levels increased with the severity of HFRS (Table 3, Figure 1); dynamic monitoring of HMGB-1 could benefit the early prediction of prognosis (Table 5, Figure 3), which indicates that HMGB-1 plays an essential role in the pathogenesis of HFRS as a proinflammatory cytokine. HMGB-1 can be considered a novel biomarker to evaluate disease severity and outcome in HFRS patients, with potential for applications in clinical practice.

It is believed that HFRS has the basic clinical characteristics of sepsis, but it also has a unique pathophysiologic feature. The hypotensive phase of HFRS (e.g., low blood pressure and circulation collapse) usually occurs between day 3 and day 7 of the clinical course, and grave HFRS patients can manifest more severe leukemoid reaction [17], plasma leakage, and coagulation disorders [18, 19] compared with septic patients, which would lead to massive bleeding, profound shock, severe tissue hypoperfusion, and severe hypoxia, potentially rendering renal, cardiac, cerebellar, and hepatic injury [4, 19, 20]. This is similar to the data shown in this study, except that HMGB-1, WBC, PLT, and ALB also demonstrated statistical significance for predicting prognosis, which can reflect the degree of inflammatory reaction, destruction, or dysfunction of PLT [21] and the loss of vascular integrity with increasing degree of vascular permeability [3, 22, 23]. In this study, Pearson's correlation analysis revealed that HMGB-1 was closely correlated with WBC, PLT, and ALB (Table 4, Figure 2), and the sensitivity and specificity of the combined HMGB-1, WBC, PLT, and ALB for predicting prognosis were better than those of single analysis (Table 5, Figure 3); this further indicates the effective role of HMGB-1 for evaluating the severity and outcome, and the use of HMGB-1, WBC, PLT, and ALB in combination to predict the outcome in patients with HFRS exhibited an acceptable diagnostic capability.

As a perspective study, we evaluated the predictive role of HMGB-1 on the severity and prognosis of HFRS, while there were still some limitations. First, this study was conducted at a single center for infectious diseases. The length of time form collection of the blood samples from the patients was not unified or precise, considering the different clinical conditions and phases on admission, and we can only define two periods, the acute and convalescent stages. Although there was no significant difference in the median collection time of the samples, the dynamic change of the levels of HMGB-1 can also be influenced by this variation. Second, the relatively small number of cases made the statistical power small relatively. There were only 12 patients who died, which would influence the result of the ROC curve analysis. Third, the clinical outcomes and classifications of the HFRS patients might be biased due to the lack of a more standardized protocol for the management of patients with HFRS until now.

## Figures and Tables

**Table 1:** Demographic characteristics for patients with HFRS.

	Mild group (*n* = 19)	Moderate group (*n* = 25)	Severe group (*n* = 27)	Critical group (*n* = 34)	Control group (*n* = 28)
^a^Male, *n* (%)	14 (73.7)	20 (80.0)	24 (88.9)	27 (79.4)	22 (78.6)
^b^Age, years	36.58 ± 16.24	37.64 ± 12.98	44.00 ± 14.41	45.71 ± 14.50	38.65 ± 13.26

^a^Pearson's *χ* ^2^ test: *χ* ^2^ = 1.998, *P* = 0.736.

^b^ANOVA: *F* = 2.275, *P* = 0.085.

**Table 2:** Time frame from disease onset to sample collection in patients with HFRS.

Length of time	Mild group (*n* = 19)	Moderate group (*n* = 25)	Severe group (*n* = 27)	Critical group (*n* = 34)
^a^Acute stage, days	6 (2)	5 (2)	5 (3)	4 (2)
^b^Convalescent stage, days	13 (4)	14 (4)	17 (8)	15 (11)

Data are presented as median (IQR).

^a^Kruskal-Wallis *H* test: *χ* ^2^ = 6.363, *P* = 0.095.

^b^Kruskal-Wallis *H* test: *χ* ^2^ = 4.872, *P* = 0.181.

**Table 3:** Levels of HMGB-1 in patients with HFRS.

	Mild group (*n* = 19)	Moderate group (*n* = 25)	Severe group (*n* = 27)	Critical group (*n* = 34)	^a^Control group (*n* = 28)
HMGB-1, ng/mL					
^b^Acute stage	7.08 (7.07)	7.86 (9.31)	12.54 (12.93)	24.50 (49.21)	1.68 (0.69)
^c^Convalescent stage	2.85 (2.90)	3.92 (2.06)	4.05 (2.93)	6.80 (4.52)	1.68 (0.69)

^a^The median level of the 28 control samples included.

^b^Kruskal-Wallis *H* test: *χ* ^2^ = 14.708, *P* = 0.002.

^c^Kruskal-Wallis *H* test: *χ* ^2^ = 20.324, *P* < 0.001.

**Table 4:** Pearson correlation analysis in patients with HFRS.

Variables	HMGB-1	
	*r*	*P* value
WBC	0.316	<0.001
PLT	−0.588	<0.001
HCT	0.071	0.357
ALB	−0.466	<0.001
BUN	0.309	<0.001
Scr	0.153	0.046
UA	−0.271	<0.001

*r*: correlation coefficient; HMGB-1: high mobility group box protein-1; WBC: white blood cells; PLT: platelets; HCT: hematocrit; ALB: albumin; BUN: blood urea nitrogen; Scr: serum creatinine; UA: uric acid.

**Table 5:** Predictive values for prognosis with HMGB-1 and laboratory parameters in patients with HFRS.

Variables	AUC	^a^ *P* value	^b^Probability value	^c^Sensitivity	^c^Specificity	^c^95% Cl for AUC	
						Lower	Upper
HMGB-1	0.800	<0.001	0.072	76.9	88.0	0.645	0.955
WBC	0.831	<0.001	0.111	61.5	91.8	0.724	0.937
^d^PLT	0.839	<0.001	0.147	76.9	79.7	0.753	0.925
^d^ALB	0.806	<0.001	0.082	76.9	79.1	0.690	0.922
BUN	0.619	0.154	—	—	—	0.484	0.754
Scr	0.552	0.532	—	—	—	0.429	0.675
^d^UA	0.661	0.054	—	—	—	0.502	0.820
^e^Combination	0.892	<0.001	0.103	84.6	83.5	0.807	0.977

AUC: area under the curve; CI: confidence interval; HMGB-1: high mobility group box protein-1; WBC: white blood cells; PLT: platelets; HCT: hematocrit; ALB: albumin; BUN: blood urea nitrogen; Scr: serum creatinine; UA: uric acid.

^a^ *P* value for calculated AUC in predicting death.

^b^Probability values were calculated by logistic regression.

^c^Sensitivity, specificity, and 95% CI are all presented as percentages.

^d^Test direction: lower test result indicates a more positive test.

^e^WBC, AST, PLT, and Fib in combination.
